# Supplementary material for: Transmission of Zearalenone, Deoxynivalenol, and Their Derivatives from Sows to Piglets during Lactation
Source: Toxins (Basel). 2021 Jan 6;13(1):37. doi: 10.3390/toxins13010037 (PMC7825292; doi:10.3390/toxins13010037)
Supplement: Supplementary file 1 [file toxins-13-00037-s001.pdf]

Supplementary Materials: Transmission of zearalenone, deoxynivalenol, and their derivatives from sows to piglets during lactation

Xandra Benthem de Grave, Janine Saltzmann, Julia Laurain, Maria A Rodriguez, Francesc Molist, Sven Dänicke and Regiane R Santos

Table S1. Composition (%) and calculated nutrient levels (g/kg) of the sows experimental diet.

INGREDIENTS		
-	Corn	40.0
	Wheat	12.1
	Sunflower seed meal	10.0
	Wheat middling's	7.24
	Soybean hulls	7.50
	Sugar beet pulp	5.00
	Molasses Cane	4.00
	Linseed	3.68
	Soybean meal	3.01
	Potato	2.23
	Palm oil	0.50
	Soybean oil	0.50
	Limestone	1.25
	Sodium bicarbonate	0.35
	Monocalcium phosphate	0.86
	Salt	0.21
	Lysine-HCL (L 79%)	0.32
	Threonine (L 98%)	0.24
	Methionine (DL 99%)	0.00
	Tryptophan (L 98%)	0.01
	Phytase Sow	0.10
	Premix Gestation	0.50
	Premix Lactation	0.40
NUTRIENTS		
g/Kg	Moisture	122.1
g/Kg	Crude Protein	150.0
g/Kg	Ash	58.8
g/Kg	Crude Fibre	70.4
g/Kg	Sugar	51.9
g/Kg	Crude Fat	46.6
g/Kg	Starch	334.1
MJ/kg	NE	2269
g/Kg	SID_LYSs	7.6
g/Kg	SID_METs	2.4
g/Kg	SID_M+Cs	4.4
g/Kg	Ca	9.3
g/Kg	P	5.7
g/Kg	Na	2.1

g/Kg	Cl	3.3
g/Kg	K	8.4
meq	dEB	213
mg/kg	Cu	23.1
mg/kg	Zn	139.6
	US ratio	4.6
g/Kg	FCHO	160.0
g/Kg	iCHO	80.0
g/Kg	NSPs	226.4

Table S2. Composition (%) and calculated nutrient levels (g/kg) of the creep feed.

**Ingredients**		**Creep feed**
	Potato protein	1.30
	Barley	16.00
	Oat flakes	10.00
	Coconut oil	1.50
	Milk powder skimmed	2.50
	Soybean oil	1.75
	Sugar Feed grade	3.20
	Wheat	18.62
	Wheat middling's	6.20
	Monocalcium phosphate	1.26
	P start 2220 0,4%	0.40
	Whey powder sweet	10.00
	Salt	0.33
	Prem. vitamin ad3e	0.10
	Premix Copper-pigs	1.74
	Maize expanded	13.33
	Soycomil (spc)	4.20
	Vitamins/trace-elem.	0.10
	Lysine-HCL (L 79%)	0.50
	Methionine (DL 99%)	0.14
	Threonine (L 98%)	0.16
	Tryptophane (L 98%)	0.05
	Wheat gluten meal	1.40
	Valine (L 99%)	0.03
	Sodium bicarbonate	0.10
	Fish oil	0.50
	Limestone	0.70
	Soya bean meal	3.90
**Nutrients**		
g/Kg	Moisture	102.29
g/Kg	Crude protein	167.10
g/Kg	Ash	53.95
g/Kg	Crude fibre	23.59
g/Kg	Sugar	135.82
g/Kg	Fat	65.42
g/Kg	Starch	342.70
MJ/kg	NE	10.21
g/Kg	SID_LYSs	11.07
	SID met/SID lys	0.35
	SID m+c/SID lys	0.58
